# The influence of internship satisfaction and the psychological contract on the career identity behavior of fresh graduates

**DOI:** 10.3389/fpsyg.2023.1294799

**Published:** 2023-12-07

**Authors:** Ying Feng, Zhuo Zhang, Xiuzhen Zeng, Yuying Liu

**Affiliations:** ^1^School of Humanities and Law, University of Science and Technology Beijing, Beijing, China; ^2^School of Economics and Management, University of Science and Technology Beijing, Beijing, China; ^3^Research & Training Centre for UNESCO Asia-Africa TVET Project, Shenzhen Polytechnic University, Shenzhen, China; ^4^Tianjin University of Commerce Cooperative School of International Education, Tianjin University of Commerce, Tianjin, China

**Keywords:** career identity behavior, internship satisfaction, psychological contract, dimensions, fresh graduates

## Abstract

**Introduction:**

Frequent resignation of young workers brings huge costs to the organizational management of enterprises. The frequent turnover behavior is a sign of low career identity, and exploring the paths that influence career identity behaviors is necessary. Previous studies have found that internship satisfaction and the psychological contract can influence career identity behavior. However, the dimensions of the psychological contract are unclear, and it is uncertain whether internship satisfaction can influence career identity behavior through the different dimensions of the psychological contract. This study attempts to expand the concept of psychological contract and construct a multiple mediation model. It aims to analyze the mediating role of different dimensions of the psychological contract between internship satisfaction and career identity behavior.

**Methods:**

A sample survey was conducted on Chinese fresh graduates by way of the questionnaire survey, and a total of 576 valid questionnaires were collected. Amos 26.0 was used to analyze the data and verify the multiple mediation model.

**Results:**

The results showed that psychological contract can be divided into three dimensions: transactional contract, relational contract, and developmental contract. Internship satisfaction can positively influence career identity behavior via the three dimensions of psychological contract, and there are differences in mediating effects among the dimensions. The mediating effect of developmental contract is the highest, relational contract is the second, and transactional contract is the lowest.

**Discussion:**

This article expands the dimensions of psychological contract, emphasizes the importance of developmental contract, contributes to the literature on organizational psychology, and provides scales and empirical evidence for future research. The analysis points out that fresh graduates with long-term development opportunities often show higher career identity behavior. This provides valuable insights for enhancing career identity behavior, improving career sustainability, and assisting organizations in managing human resource mobility.

## 1 Introduction

In recent years, the economy has been hard hit by the COVID-19 pandemic and regional conflicts (Warwick and David, 2020; Chisadza and Clance, 2021; Mahmood et al., 2022; Taylan et al., 2022). In such a period of economic downturn, business bankruptcies increase and job opportunities decrease. For the normal order of life not to be disrupted, people tend to seek more stable jobs (Sánchez-Sellero et al., 2017). However, young people are doing the opposite, as the rate of active job change has not declined (Mycos Research Institute, 2022). This behavioral trend reflects a lack of career identity (Bondt and Vermeulen, 2020; Cerra et al., 2023). The high employee turnover rate is a marked cost burden on enterprises and has become one of the bottlenecks impeding their development. Therefore, we investigated the factors and paths influencing career identity behavior, which are of guiding significance for career sustainability, enterprise organizational management and enterprise development. Due to China’s farming culture and Confucianism, the Chinese prefer stability (Shih, 2014; Liu et al., 2016). However, there has been a high separation rate among younger workers in recent years. In 2022, more than 30 percent of fresh graduates in China left their jobs within 6 months (Mycos Research Institute, 2022). These statistics illustrate a lack of career identity behavior among the youth. Thus, conducting a study on this shift in behavior using fresh graduates in China as an example would be representative and valuable conducting.

Career identity behavior refers to an individual’s attitude and performance toward the occupational environment and role in which they work. This concept includes one’s recognition and belonging to the job. According to career identity behavior, we can evaluate an individual’s work engagement and loyalty (Obling, 2022; Weiss et al., 2023). However, despite its importance, the career identity behavior of fresh graduates remains an understudied domain, resulting in limited empirical research to draw from. Understanding the factors that influence the development of career identity behavior has wide-reaching implications for the facilitation of optimal learning experiences and the overall productivity of the workforce (Cheung and Arnold, 2014).

Drawing on social identity theories, internship satisfaction among fresh graduates is important for the formation of their career identity (Willetts and Clarke, 2013; Cai et al., 2022; Wang and Binti Omar, 2023). High internship satisfaction is often associated with greater engagement, motivation and commitment, and contributes to a sense of career identity (Seyitoğlu and Yirik, 2014; Qu et al., 2021). Moreover, scholars have found that a satisfying internship can foster a positive psychological contract. Psychological contract is a series of intangible and implicit expectations that exist between organizations and individuals (Fantinelli et al., 2023), which greatly affects the behavior of employees and the organizational management of enterprises. For example, Interns who have a fulfilling learning experience, feel valued, and perceive fair treatment are more prone to develop a strong psychological contract with their organization (D'Abate, 2009; Suazo and Turnley, 2020; Simons, 2021). Conversely, when the psychological contract is broken, betrayal and resentment may arise, which negatively impacts productivity and morale (Bal et al., 2010). Human behavior is a controllable system, and it makes sense to study this organizational behavior with the help of psychological contracts. Organizational psychology suggests that the fulfillment of psychological needs directly affects one’s career identity (Trede et al., 2012; Stuer et al., 2019). Although the influencing factors of career identity behavior and dimensions of the psychological contract have been studied in the existing literature, it is not currently clear whether internship satisfaction can stimulate career identity behavior through different dimensions of the psychological contract. This study attempts to extend the concept of the psychological contract and analyze the mediating roles of its different dimensions between internship satisfaction and career identity behavior, so as to enhance career sustainability.

This research analyzes whether internship satisfaction can influence the career identity behavior of fresh graduates through different dimensions of the psychological contract. They often gain a sense of the internship after participating in it, which leads to a clearer perception and vision of their career path (Trede et al., 2012). The higher the career identity, the higher the career sustainability. This paper attempts to address the following theoretical questions:

Q1: Does the psychological contract develop new connotations and dimensions with the development of the times?

Q2: Do the three dimensions of the psychological contract mediate the link between internship satisfaction and the psychological contract?

Q3: How do the three dimensions of the psychological contract differ in their mediating role on career identity behavior?

Our study aims to provide the following contributions to the existing literature. Firstly, the paper refines the understanding of the psychological contract by dividing it into three dimensions. In addition, the existing scales have been innovatively optimized to align with the research topic, thereby providing instrumental support for empirical research in related areas. Secondly, we examine the mediating effect of the three dimensions of the psychological contract between internship satisfaction and career identity behavior, which built an unprecedented bridge between internship satisfaction and career identity behavior. We also analyze the differences in the mediating effects of psychological contract and attempt to fill the gap of organizational psychology. This study can provide guidance for the organizational management of enterprises by implementing the innovative model, and address the issues of human resource stability and sustainable development of enterprises.

## 2 Literature review and research hypotheses

### 2.1 Psychological contract

The concept of the psychological contract was introduced by the social psychologist Argyris to describe a non-obvious and informal mutual relationship between employers and employees. He found that employees’ productivity increased when they perceived that the company would provide benefits to them beyond the obligations specified in the written contract (Argyris and Ditz, 1960; Topa et al., 2022; Fantinelli et al., 2023). The connotations and dimensions of the psychological contract have been studied, to some extent by previous scholars. In general, proponents of the binary structure of the psychological contract argue that it should be divided into a transactional contract and a relational contract (Rousseau, 1989; Robinson and Rousseau, 1994; Tsui et al., 1997). Rousseau also introduced the concept of balance, which mixed financial or material components with social and symbolic components, such as performance-reward contingencies (Rousseau, 1995, 2004; Mehta et al., 2022). Li′s empirical study of 796 corporate employees identified the existence of a developmental psycho-logical contract that varies across different cultures (Li, 2002). The connotation of the psychological contract changes with the social environment. With the emergence of new economic forms such as gig economy and smart economy, when organizations are unable to promise long-term and stable employment relationships to employees, the employees’ demand for self-improvement will become strong. Moreover, people are increasingly pursuing the development of their own potential, and the traditional connotation of the psychological contract does not focus on these. Therefore, social change has given more meaning to the psychological contract. However, the current study is not enough in this area. This study classifies the dimensions of the psychological contract into a transactional contract, relational contract, and developmental contract. These three dimensions are closely related to each other yet conceptually interdependent, and it is this theoretical gap that our study intends to fill. The concept of the psychological contract was refined into a second-order measure to gain a more specific meaning.

First, a transactional contract (TRC) represents a functional interaction based on short-term material benefits (Robinson and Rousseau, 1994). It generally includes the economic and material conditions provided by the company, including one’s salary, benefits, and working environment. This dimension has stable and visible properties.

Second, a relational contract (REC) focuses on broader and long-term social emotional interaction at the spiritual level. It encompasses the interpersonal atmosphere created by the enterprise for its employees (Rousseau, 1989; Tsui et al., 1997; Shen et al., 2019), including the respect and care of its employees, the recognition of employees’ contributions and support from superiors. This dimension has dynamic and intangible properties.

Third, a developmental contract (DEC) signifies that employees have the resources and opportunities for career development at work (De Vos et al., 2020), so that they can develop their strengths and potential (Li, 2002). This type of contract incorporates elements such as training, career progression, and other opportunities that help the employees to grow and evolve in their careers. This dimension has sustainable and long-term properties.

### 2.2 The psychological contract as a mediator

Social exchange theory posits that reciprocal relationships constitute a dynamic mechanism in social development. Reciprocity is the exchange of social psychology and social behavior in interpersonal relationships, and the cost of the exchange is not limited to material wealth, but also includes psychological wealth (Simbula et al., 2023). The relationship between an organization and its employees, as governed by the psychological contract, represents a reciprocal exchange. Unlike economic exchanges, which are typically based on explicit regulations, this exchange occurs through the mental measurement of the individual and a comparison based on social norms and values. Employees may be more satisfied and committed if they perceive that their psychological contract is being met (e.g., they receive the promised learning opportunities, fair treatment, and support). This positive emotional connection can enhance their willingness to continue in the profession and their career identity behavior (Rayton and Yalabik, 2014; Bellini et al., 2022).

In addition, during the internship, employees compare their practical experiences with their psychological expectations in the form of “learning by doing.” Subsequently, a psychological contract is formed when they realize that their contribution is mutually beneficial to the organization (Praskova et al., 2015; Jung, 2020). Previous studies have examined the influence of the psychological contract on career identity behavior and the influence of internship satisfaction on the psychological contract, but there is not yet research on the psychological contract and how it mediates the link between internship satisfaction and the psychological contract. The following demonstrates that internship satisfaction influences career identity behavior through three dimensions of the psychological contract (TRC, REC, DEC).

#### 2.2.1 The mediating role of the transactional contract

The transactional contract is characterized by a concrete and visible reciprocal exchange, which can be affected by perceived internship satisfaction. Employees who are satisfied with their internship will feel a fair exchange of benefits, e.g., they give their skills and time and receive financial compensation in line with their psychological expectations. Conversely, the transactional contract breaks down if employees are dissatisfied with how they feel they are being treated during the internship, as they may feel that they are not receiving adequate remuneration for their input (Tomprou et al., 2015).

**Figure 1:**
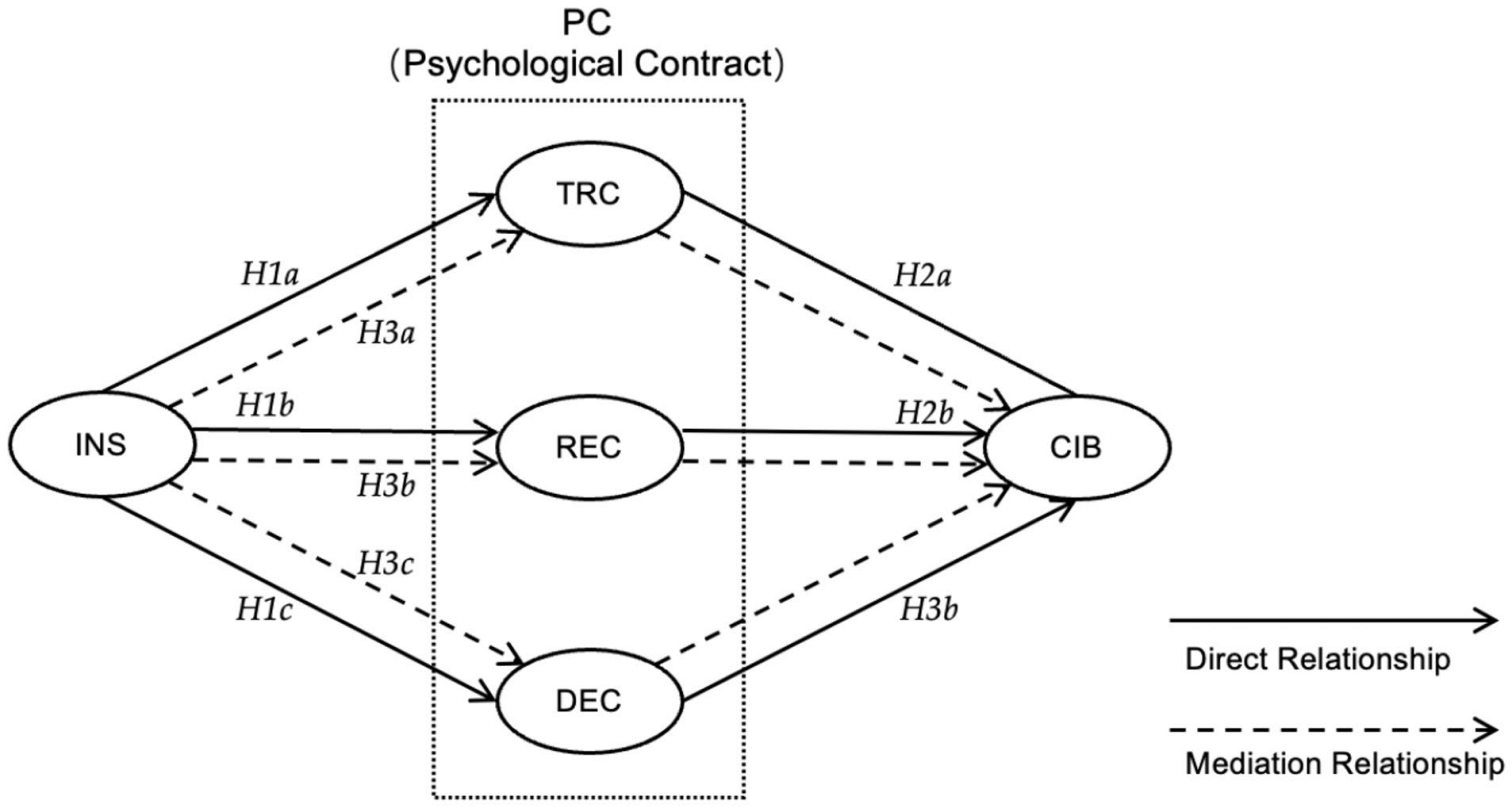
Theoretical model.

The transactional contract affects career identity behavior because it is typically tied to specific deliverables or goals (Mehta et al., 2022). This connection is associated with clearly defined performance metrics and is an important driver for stimulating self-performance, which can positively influence organizational identity, and then improve career identity (Liu et al., 2020; Rogozińska-Pawełczyk and Gadomska-Lila, 2022). As productivity increases, pay levels are typically stimulated, further reinforcing the transactional contract. While the level of pay is a critical element in assessing whether to continue in a job, satisfaction with the transactional contract plays a vital role in shaping one’s career identity behavior (De Hauw and De Vos, 2010; Dries et al., 2014). Accordingly, we propose the following hypothesis (Figure 1):

*Hypothesis 1a (H1a)*. Internship satisfaction (INS) has a significant positive effect on the transactional contract (TRC).

*Hypothesis 2a (H2a)*. The transactional contract (TRC) has a significant positive effect on career identity behavior (CIB).

*Hypothesis 3a (H3a)*. The transactional contract (TRC) mediates the impact of internship satisfaction (INS) on career identity behavior (CIB).

#### 2.2.2 The mediating role of the relational contract

The relational contract goes beyond typed reciprocity, and delves into the realm of emotional connection, which can be affected by internship satisfaction. Employees who are satisfied with their internship experience are more likely to develop a higher level of trust in the organization. They may also be more open to communicating with their supervisors and peers. This open communication can help maintain and strengthen the relational contract by facilitating a mutual understanding and addressing any potential issues or misunderstandings (Rousseau, 1989; McDermott et al., 2013). This heightened engagement can strengthen the relational contract as it is often accompanied with a sense of belonging and connection to the organization.

The relational contract influences one’s career identity behavior. This type of contract plays a critical role in shaping the employee’s perception of their role within the organization. The contract breach discourages employees from investing in the organization. Conversely, covenant fulfillment creates a sense of belonging to the occupational community and fosters alignment with the organization’s culture (Liu et al., 2020). which means they might be more inclined to engage in discretionary efforts, go beyond their strict role requirements, and show a commitment to the organization. With the establishment of a relational contract, interns may be more likely to return to full-time positions or recommend the organization to others (Ng et al., 2010). This type of contract helps to reduce turnover, which, in turn, enhances career identity behavior. Accordingly, we propose the following hypothesis (Figure 1):

*Hypothesis 1b (H1b)*. Internship satisfaction (INS) has a significant positive effect on the relational contract (REC).

*Hypothesis 2b (H2b)*. The relational contract (REC) has a significant positive effect on career identity behavior (CIB).

*Hypothesis 3b (H3b)*. The relational contract (REC) mediates the impact of internship satisfaction (INS) on career identity behavior (CIB).

#### 2.2.3 The mediating role of the developmental contract

The developmental contract is affected by the intern’s perceived satisfaction with their internship. Internship satisfaction usually comes from gaining practical skills and recognition. When interns are satisfied with their experience, they are more likely to perceive the company as offering valuable career development opportunities. As technology and the labor market are changing rapidly, interns expect learning and development opportunities from the organization (Lodi et al., 2020). This perception contributes to the formation of a positive developmental contract, wherein interns believe that the organization is committed to their long-term growth. From an organizational perspective, when interns perform well and make meaningful contributions, organizations are also more likely to invest in their development, thereby reinforcing the developmental contract (Li, 2002; Bal et al., 2013).

The developmental contract involves providing employees with opportunities and platforms to develop themselves and align their personal goals and values with their occupational roles and responsibilities. This alignment has the potential to strengthen career identity. By providing opportunities for individuals to interact with mentors or role models, organizations offer guidance and inspiration to interns and help individuals gain a deeper understanding of their career and their role within it. This deep investment in the employee’s future and can facilitate clear career development goals for employees, and lead to a range of positive outcomes for both the employees and the organization (Ng et al., 2010; Coyle-Shapiro et al., 2019). As they develop their skills and sustainable advance in their careers, they can experience a sense of accomplishment and increased confidence in their occupational abilities. This can enhance their occupational self-esteem, which is a key component of career identity behavior (Figure 1).

*Hypothesis 1c (H1c)*. Internship satisfaction (INS) has a significant positive effect on the developmental contract (DEC).

*Hypothesis 2c (H2c)*. The developmental contract (DEC) has a significant positive effect on career identity behavior (CIB).

*Hypothesis 3c (H3c)*. The developmental contract (DEC) mediates the impact of internship satisfaction (INS) on career identity behavior (CIB).

## 3 Methodology

### 3.1 Research approaches and procedures

First, we used the questionnaire method as the research tool to get primary data from fresh graduates. Second, we established measures for the constructs to transform qualitative attributes into a form suitable for statistical analysis. Third, we conducted common method variance tests to identify and mitigate potential distortions in the data. Then, we scrutinized the questionnaire’s reliability and validity through confirmatory factor analysis, discriminant validity, and model fit tests, affirming its suitability for structural equation modeling. The final phase of this research process was empirical testing (Yang et al., 2023). In this phase, we employed structural equation modeling, applying the developed model to real-world data to test the hypotheses in an empirical context. Our article used Amos 26.0 to test the multiple mediation effect model and to compare the mediation effects. In addition, we used the method of bootstrap to simulate the sampling distribution process with 5,000-times, which made the findings more statistically significant.

### 3.2 Sampling and data collection

Our research obtained the data by a questionnaire. In order to examine the relationship between internship satisfaction, psychological contract, and career identity behavior, we selected fresh graduates with internship experience. A total of 650 questionnaires on internship satisfaction, psychological contract and career identity behavior, were distributed by a combination of personal visits, letters, and online distribution (Gresse and Linde, 2020). After excluding 23 questionnaires with incomplete/invalid responses, 576 valid questionnaires were obtained, with a valid response rate of 88.6%.

The sample was extensive and covered various regions across China, including the economically developed southeastern coastal provinces, such as Guangdong, Jiangsu, and Shandong, as well as the economically less developed inland provinces, such as Shaanxi, Sichuan, and Hebei. In the valid sample, male accounted for 58%, female accounted for 42%, and the participants were predominantly 20–24 years old, of which 22 years old had the highest percentage (67%). There was a wide distribution of majors among the fresh graduates, with a high percentage of them majoring in mechanical engineering and automation. Also, to ensure the authenticity of the questionnaires completed by the respondents, we clearly communicated the academic purpose and value of the study to them. We also assured that the questionnaires would be filled out and processed anonymously (Gligor et al., 2015; Tortorella et al., 2019).

### 3.3 The construct measures

The measurement of the constructs involved translating the theoretical concepts into quantifiable variables, rendering them amenable to statistical analysis. The questionnaire items for this research were adapted from validated scales in the literature or specifically formed scales based on previously proposed metrics. We adapted the scales where necessary to the topic of this study and in conjunction with experts’ opinions (see Table 1). The scales were presented on a 5-point Likert scale, with responses ranging from 1, indicating strong non-conformity, to 5, indicating strong conformity.

**Table 1:** The constructs and measurement items.

Item	Loading	CR	AVE	Cronbach’s alpha
Internship satisfaction (INS)				
Internship planning (INS1)	0.798***	0.896	0.632	0.895
Internship assignment (INS2)	0.774***			
Educational training (INS3)	0.766***			
Administrative support and assistance (INS4)	0.808***			
Performance evaluation (INS5)	0.827***			
Transactional contract (TRC)				
A fair salary (TRC1)	0.813***	0.913	0.676	0. 913
A competitive salary (TRC2)	0.825***			
Pay tied to my level of performance (TRC3)	0.818***			
Stable job security (TRC4)	0.845***			
Nice working environment (TRC5)	0.811***			
Relational contract (REC)				
Harmonious working atmosphere (REC1)	0.800***	0.930	0.728	0.930
The extent to which I am treated with respect (REC2)	0.848***			
The extent to which I am treated fairly and impartially (REC3)	0.868***			
The amount of support I receive from management (REC4)	0.867***			
Recognize my contribution to the company (REC5)	0.881***			
Developmental contract (DEC)				
Work is challenging (DEC1)	0.783***	0.917	0.689	0.917
Provide opportunities for career development (DEC2)	0.820***			
Opportunity for learning and training (DEC3)	0.846***			
Have autonomy at work (DEC4)	0.838***			
Allow me to use my strengths (DEC5)	0.861***			
Career identity behavior (CIB)				
Positive to face career frustration (CIB1)	0.800***	0.924	0.709	0.923
Believe the job is socially acceptable (CIB2)	0.834***			
Show self-identification (CIB3)	0.862***			
Show recognition of occupational skills (CIB4)	0.847***			
Show recognition of occupational role (CIB5)	0.864***			

***p < 0.001.

#### 3.3.1 Internship satisfaction

We adapted a well-established scale from the existing literature to create the independent variable internship satisfaction (INS) scale for this study (Chen et al., 2018). This scale consisted of five items that included both fresh graduates’ attitudes toward internship arrangements and their views of their internship units. Specifically, it included internship planning, internship assignments, educational training, administrative support and assistance, and performance evaluation. After conducting a reliability assessment, the Cronbach’s alpha was 0.895.

#### 3.3.2 Psychological contract

According to previous analyses, the mediating variable, the psychological contract (PC)was divided into three dimensions: the transactional contract, the relational contract, and the developmental contract. These three scales were formed based on this division (Robinson and Wolfe Morrison, 2000; Li, 2002; Turnley, 2003). By adapting the existing scales, each of the three scales in this study contained five items.

First, the transactional contract (TRC) outlines the concrete aspects of an employment relationship, including appropriate compensation that reflects one’s performance, job stability, and a pleasant physical workspace. The Cronbach’s alpha coefficient of the scale was 0.917.

Second, the relational contract (REC) embodies the emotional and interpersonal elements of work, focusing on the creation of a respectful, fair, and supportive environment where individual contributions are acknowledged and appreciated. The Cronbach’s alpha coefficient of the scale was 0.930.

Third, the developmental contract (DEC) focuses on career preparation for the future, emphasizing the challenging tasks, career advancement opportunities, continuous learning, workplace autonomy, and the ability to utilize one’s strengths. The Cronbach’s alpha coefficient of the scale was 0.913.

#### 3.3.3 Career identity behavior

The dependent variable, career identity behavior, was modified from existing scales. The scale consists of five items that address the self-identified behaviors of careers and the social performance of career identity. Specifically, it is expressed as: being positive in facing career frustrations, believing the job is socially acceptable, showing self-identification, showing recognition of occupational skills, and showing recognition of the occupational role. The reliability test showed that the Cronbach’s alpha coefficient of the scale was 0.923.

### 3.4 Common method variance

To effectively prevent common method bias (see Table 2), we adopted multiple approaches (Baron and Kenny, 1986; Podsakoff et al., 2003; Poppo et al., 2016; Dubey et al., 2018). First, we used Harman’s single-factor test to check the model fit of the one-factor model. The poor fit indicated that there was no obvious common method bias. Second, we conducted a comparative analysis with the common method factor model, and found no obvious difference, which further confirmed that the common method bias was small. Consequently, we concluded that common method bias in this study was relatively minor.

**Table 2:** Common method bias.

	RMSEA	PNFI	CFI	NFI	IFI	TLI	x^2^/df	x^2^	df
Original model	0.043	0.839	0.973	0.949	0.973	0.970	2.043***	541	265
Single-factor model	0.197	0.364	0.405	0.396	0.406	0.353	23.373	6,450	276
Common method factor model	0.039	0.769	0.980	0.958	0.980	0.975	1.867	450	241
Criteria	<0.08	>0.5	>0.9	>0.9	>0.9	>0.9	<3		

RMSEA, root mean square error of approximation; PNFI, parsimonious normed fit index; CFI, comparative fit index; NFI, normed fit index; IFI, incremental fit index; TLI, tucker-Lewis index; x^2^/df, the ratio of chi-square to degrees of freedom.

### 3.5 Validity and reliability

We used a combination of the following approaches for validity and reliability testing. In the first stage, the reliability of the items was determined by checking the loading. If the loading was lower than 0.60, it indicated a weak correlation between the item and the construct to which it belonged (Kock, 2015). All items in this study had loadings that were above 0.6, which showed that the performance of the scale is reliable (Kholaif and Ming, 2022). Additionally, Cronbach’s alpha and composite reliability (CR) are essential indicators of reliability, with items above 0.7 meeting the requirements (Fornell and Larcker, 1981; Kline, 2016). The values for all items in the research were higher than 0.7 threshold (see Table 1).

In the second stage, we tested convergent validity and discriminate validity through confirmatory factor analysis. Specifically, Convergent validity assesses whether each indicator reflects the same construct. If the convergent validity is low, it suggests that the indicators reflect different constructs and meanings. As evident in Table 1, good convergent validity is indicated when the average variance extracted (AVE) value exceeds 0.5, and the composite reliability (CR) value exceeds 0.7 (Fornell and Larcker, 1981; Kline, 2016). Discriminative validity is also one of the ways to test validity (Yang et al., 2022). If the correlation coefficient is less than the square root of the AVE, it passes the test. As shown in Table 3, the values of the research items in this study met the above requirements, indicating satisfactory discriminant validity.

**Table 3:** Correlations and discriminate validity.

	AVE	CIB	TRC	DEC	REC	INS
CIB	3.141	0.795				
TRC	3.525	0.364	0.830			
DEC	3.492	0.490	0.306	0.853		
REC	3.292	0.452	0.330	0.335	0.822	
INS	3.693	0.415	0.263	0.297	0.257	0.845

All the correlations were significant at *p* < 0.01.

In the third stage, we checked the model fit of the structural equation model (SEM; Hu and Bentler, 1999; Chacón-Cuberos et al., 2019). The values of the indicators were within the acceptable range, indicating that the items of the scale were reasonably set and suitable for structural equation analysis (χ^2^/*df* = 2.368, RMSEA =0.049, PNFI = 0.840, IFI = 0.945, NFI = 0.941, CFI = 0.965, TLI = 0.960).

## 4 Hypothesis testing

### 4.1 Direct effects

**Figure 2:**
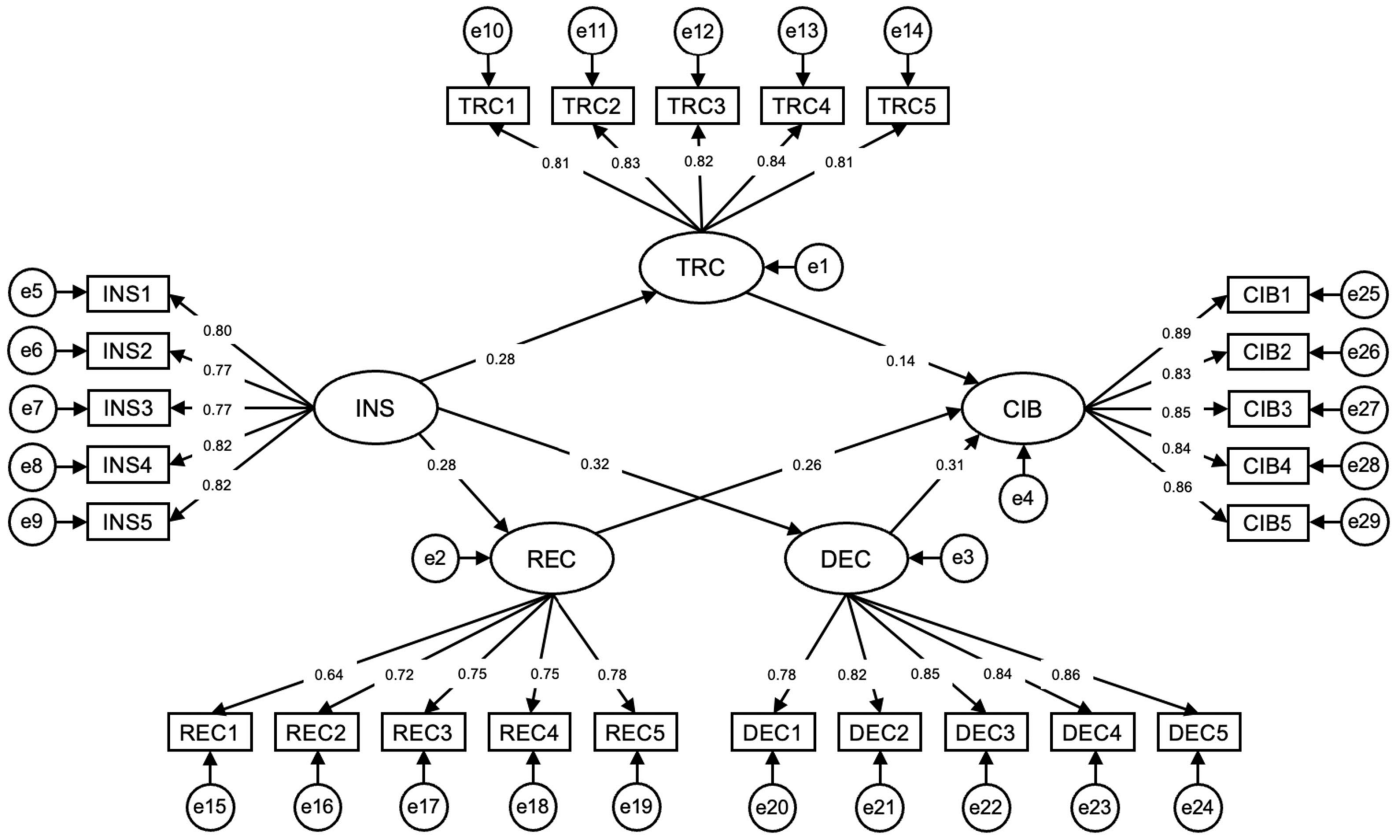
Structural equation model.

SEM was used to directly test the effects and evaluate the hypotheses outlined above (Yang et al., 2023). For each case, the provided results include Standardized Beta (Standard Beta, Std. beta), Standard Error (Standard Error, SE), and *p*-values (Shrout and Bolger, 2002). The results show that the hypotheses all successfully passed the testing (see Figure 2 and Table 4).

**Table 4:** Path relationships of the direct effects.

Hypothesis	Path	Std.beta	SE	P
H1a (supported)	INS to TRC	0.280	0.046	***
H1b (supported)	INS to REC	0.275	0.042	***
H1c (supported)	INS to DEC	0.312	0.043	***
H2a (supported)	TRC to CIB	0.141	0.029	***
H2b (supported)	REC to CIB	0.263	0.032	***
H2c (supported)	DEC to CIB	0.311	0.033	***

INS, internship satisfaction; TRC, transactional contract; REC, relational contract; DEC, developmental contract; CIB, career identity behavior. ***p < 0.001.

H1a verifies the significant effect of INS on the TRC. The standardized beta coefficient is 0.280 indicates a substantial positive relationship. The relatively small standard error (SE) of 0.046 ensures the precision of this estimate, while the three asterisks represent p-values of less than 0.001, providing strong support for statistical significance. Similarly, H1b and H1c verify that INS has a positive effect on REC and DEC, with standardized beta coefficients of 0.275 and 0.312, respectively. Both relationships maintain high statistical significance (*p* < 0.001), reflecting a positive effect of INS on all three dimensions of the psychological contract (TRC, REC, DEC).

Similarly, H2a, H2b, and H2c examined the direct impact of TRC, REC, and DEC on CIB, respectively. The beta coefficients range from 0.141 to 0.311, showing varying degrees of positive relationships. All relationships maintain consistent statistical significance (*p* < 0.001), verifying the positive influence of all three dimensions of the psychological contract (TRC, REC, DEC) on CIB.

### 4.2 Mediation analysis

As shown in Table 5. The effect of internship satisfaction (INS) on career identity behavior (CIB) can be observed through three paths: the transactional contract (TRC), the relational contract (REC), and the developmental contract (DEC). To test the mediating effect stated in the hypothesis, this study employed bootstrapped mediation (Preacher and Hayes, 2004; Bracho-Amador et al., 2023; Granero-Gallegos et al., 2023). With a Bootstrap of 5,000, all three mediating paths showed statistical significance, and the confidence intervals of the indicators did not include 0. This demonstrates the robustness of the study’s results.

**Table 5:** Mediation effect results.

					Bootstrapping			
					Bias-corrected 95% CI		Percentile 95% CI	
Hypothesis	SIE	Std.beta	SE	Z	Lower	Upper	Lower	Upper
H3a(supported)	INS to TRC to CIB	0.029^***^	0.011	2.636	0.011	0.056	0.010	0.054
H3b(supported)	INS to REC to CIB	0.052^***^	0.014	3.714	0.030	0.086	0.028	0.082
H3c(supported)	INS to DEC to CIB	0.070^***^	0.016	4.375	0.044	0.105	0.042	0.103

***p < 0.001.

As for hypothesis H3a, we found that the effect of INS on CIB through TRC was significant. Specifically, the Standardized Indirect Effect (SIE) is 0.029, the Standard Error (SE) is 0.011, and the z-score is 2.636. In addition, both the 95% confidence intervals did not contain zero and ranged from [0.011, 0.056] and [0.010, 0.054], respectively. These results further confirm the significance of the mediating effect. Similarly, hypothesis H3b was tested, revealing that INS has a significant effect on CIB through REC and DEC, respectively. The SIE values are 0.052 and 0.070, SE is 0.014 and 0.016, and the Z scores are 3.714 and 4.375, respectively. Likewise, none of the 95% confidence interval ranges contain 0, confirming the significance of the mediating effect.

### 4.3 Mediation effects comparison

Based on Table 5, we realize that among the three mediating variables, DEC (0.070) has the largest mediation effect of INS on CIB, followed by REC (0.052), and TRC (0.029) has the least influence. Table 6 shows the comparison of the SIE differences among the three mediation paths. We compare mediation effects, and the difference between DEC and TRC is 0.041, the difference between DEC and REC is 0.018, the difference between REC and TRC is 0.023. In addition, both the 95% confidence intervals did not contain zero, which indicates the difference is significant.

**Table 6:** Mediation effects comparison.

				Bootstrapping			
				Bias-corrected 95% CI		Percentile 95% CI	
SIE	Std.beta	SE	Z	Lower	Upper	Lower	Upper
SIE diff comparison DEC - TRC	0.041^***^	0.005	1.739	0.033	0.049	0.032	0.049
SIE diff comparison DEC -REC	0.018^***^	0.002	0.661	0.014	0.019	0.014	0.021
SIE diff comparison REC-TRC	0.023^***^	0.003	1.078	0.019	0.030	0.018	0.028

***p < 0.001.

## 5 Discussion and implications

### 5.1 Discussion

The paper explored the pathways that influence career identity behavior. We developed and validated a multiple mediation model that reflected the relationship among internship satisfaction, the three dimensions of the psychological contract, and career identity behavior. It was found that internship satisfaction can positively influence career identity behavior via the three dimensions of the psychological contract.

We identified three dimensions of the psychological contract based on a multidisciplinary literature study. The first two dimensions (TRC, REC) align with previous research (Rousseau, 1989; Robinson and Rousseau, 1994; Tsui et al., 1997). Additionally, considering the future development needs of fresh graduates and career sustainability, we introduced a third dimension of the psychological contract named developmental contract (DEC). Unlike previous studies, our research takes into account the current social and employment context, where technological advancements have led to increased career transitions. Even artificial intelligence has replaced some jobs, and thus employees especially value the organization’s commitment to enhancing their capabilities, which will benefit the long-term development of their careers. The job market is changing rapidly and flexible. Therefore, fresh graduates are particularly concerned about the availability of sustainable development opportunities provided by companies.

The developmental contract (DEC) can improve the dynamic adaptability of fresh graduates, so that they can adapt to the changing work task. The result revealed the importance fresh graduates place on self-development in shaping the psychological contract. This perspective challenges the traditional binary structure and provides a new perspective for the understanding of the psychological contract, and enriches its explanatory framework. Subsequently, we conducted a mediation analysis based on the three dimensions of the psychological contract, enhancing the scientific rigor and precision of our research findings.

Furthermore, this study empirically investigated fresh graduates in China, a predominantly manufacturing country. Based on the results of H1a, H1b, and H1c, internship satisfaction positively influenced all three dimensions of the psychological contract (TRC, REC, DEC); and based on the results of H2a, H3b, and H3b, all three dimensions of the psychological contract positively influenced career identity behavior. This is consistent with previous studies (Rayton and Yalabik, 2014; Stuer et al., 2019). Unlike previous studies, however, our research constructed and tested a mediating effects model for the above variables. According to the findings of H3a, H3b, and H3c, the three dimensions of the psychological contract play multiple mediating roles between internship satisfaction and career identity behavior. These findings are based on previous research and emphasize the important role of the three dimensions of psychological contract in building a bridge between internship satisfaction and career identity behavior. According to social exchange theory, when the psychological contract of a fresh graduate remains unviolated during an internship, it contributes to the establishment of their career identity behavior.

Specifically, DEC has the highest mediating effect value, followed by REC, and TRC has the lowest. This pattern aligns with the attributes of the three psychological contracts. DEC pertains to the training and development opportunities provided by companies to their employees. In recent years, rapid technological advancements, including the advent of artificial intelligence, have led to job displacement, intensifying career dissatisfaction (Xu et al., 2021). Moreover, as the economic level rises and people’s survival needs are being met, people pay more attention to self-improvement. Fresh graduates may be more eager to take advantage of career development opportunities and resources to improve their skills so that they can remain competitive in the changing workplace environment. Providing employees with adequate training, career sustainable development, and other growth opportunities may be more conducive to enhancing their career identity behavior (Bal et al., 2013), and thus the reasons why DEC has the highest mediating effect.

The REC involves social and emotional interactions that can help fresh graduates cultivate a stronger sense of belonging and career identity behavior. A well-established organizational feedback mechanism helps to promote positive communication within the organization. While timely communication between superiors and subordinates can temporarily address negative emotions in the workplace, it may not be sufficient to support long-term career development and sustainable growth (Rousseau, 1989). This factor may contribute to the lower mediating effect of REC compared to DEC.

The TRC focuses on short-term financial benefits (Tomprou et al., 2015), which play a fundamental role in fresh graduates’ career identity behavior. It is crucial to provide financial support. However, it has the smallest mediating effect value, which might be due to the inconsistency between material rewards and intrinsic motivations, such as achievability and self-actualization.

### 5.2 Theoretical implications

The theoretical contributions of this study are as follows. Firstly, it enriches the understanding of the psychological contract by providing a multidimensional perspective, contributing to the literature on organizational psychology. It moves beyond the traditional binary structure of the psychological contract and especially focuses on the developmental nature of careers, which provides a theoretical basis for sustainable career development. This three-dimensional approach contributes to a more comprehensive understanding of the meaning and role of the psychological contract in the present context. This suggests that the psychological contract is not a fixed concept but can be dynamically adapted to specific cultural contexts and social situations. In the case of frequent turnover of young workers, the dimension of developmental contract is particularly important. The division of dimensions is not only for the present, but also for the future, which reflects that paying attention to the long-term development of fresh graduates is helpful to improve career identity behavior. It affects career sustainability to a great extent, and the results of empirical research also verify this view. This finding deepens our understanding of the complexity of the psychological contract, which not only provides a richer framework for understanding and explaining the psychological contract, but also provides a new basis for subsequent research.

Secondly, this study verifies the influence path of career identity behavior. The paper contributes to the field by constructing reliable indicators for internship satisfaction, the three dimensions of the psychological contract, and career identity based on social exchange theory and previous research. These provide a reliable tool for future research. Based on this, we analyze the mediating role of the three dimensions of psychological contract between internship satisfaction and career identity behavior, which provide a new perspective for theoretical development in related fields. Previous studies have focused on the relationship between internship satisfaction and career identity behavior, or the psychological contract and career identity behavior (Argyris and Ditz, 1960; Tomprou et al., 2015), but have neglected the association among these three. This study bridges this gap in research by analyzing the mediating role of the psychological contract and analyzing the mechanisms by which internship satisfaction influences career identity behavior through the three dimensions of the psychological contract. The results of the study can provide theoretical guidance to enhance the career identity behavior of fresh graduates and contribute to the stability of enterprise organizational management. Furthermore, the empirical data collected from China, a prominent manufacturing country, enhances the study’s credibility and provides empirical support for research in related fields.

### 5.3 Practical implications

The study has the following practical implications. At the macro level, the analysis of multiple mediating effects provides a fresh perspective for companies to comprehend and explain the relationship among internship satisfaction, psychological contract, and career identity behavior. The internship experiences of the employees and the resulting psychological changes can influence career identity behavior. This serves as a valuable reference for corporate internship management and organization management. For example, Companies should implement personalized welfare programs. Provide employees with personalized welfare options based on the importance they place on survival needs, relationship needs and development needs; in addition, companies should establish a flexible salary system, and employees’ salaries and wages should be linked to performance. Adopting a flexible wage system, the performance of employees should be evaluated in a fair and objective manner. Employees who feel the rewards of their internship are satisfactory are more likely to stay in the position over the long term. Therefore, companies and schools should prioritize reciprocity with fresh graduates, improve feedback and performance evaluation systems, and create a positive organizational culture. Following the internal logic of “organizational identity - self-identity - career identity” can help to improve career identity behavior. Fresh graduates with high career identity tend to be more loyal to the organization and have career sustainability. Also, it is conducive to the long-term operational development of the company and the sustainable development of the regional economy. Lower turnover rates are also good for local security.

Specifically, all three dimensions of the psychological contract play a mediating role in the relationship between internship satisfaction and career identity behavior. Therefore, more attention needs to be paid to the long-term career development of employees, building a smooth communication and feedback mechanism, and establishing a fair salary system. Considering the differences in the mediating effects of the three dimensions of the psychological contract, enterprises should pay special attention to the developmental contract and give more consideration to fresh graduates’ long-term development needs in the school-enterprise cooperation and internship process (Coyle-Shapiro et al., 2019). For example, in providing good vocational training, developing a clear career development roadmap, and providing more career development opportunities for employees. This will better meet fresh graduates’ psychological contractual expectations, improve job performance and loyalty, and thus enhance career identity behavior. Also, the company should optimize communication channels and provide humanistic care for its employees. A sound organizational culture can make up for the deficiency of other incentive measures and eliminate the psychological dissatisfaction of employees. This helps employees align their self-worth with corporate development, which in turn enhances career identity behavior and reduces turnover.

## 6 Conclusion and future directions

This study analyzed the impact of internship satisfaction and the psychological contract on the career identity behavior of fresh graduates. Based on the current situation and the Chinese context, we first explored the dimensions and meanings of the psychological contract, and classified the psychological contract into three dimensions: a transactional contract (TRC), a relational contract (REC), and a developmental contract (DEC). Subsequently, we constructed a multiple mediation model using social exchange theory and put forward the hypothesis that internship satisfaction can positively influence career identity behavior via the three dimensions of the psychological contract, and that there are differences in the mediation effects (DEC > REC > TRC). The paper draws on organizational psychology and social exchange theory to analyze the career identity behavior of fresh graduates, which provides a new idea for solving the problem of unsustainable careers and employee turnover in enterprises.

The article has certain research implications, but there are also limitations that need to be further explored based on this study. First, as an empirical study, the sample data were subjective and confined to a specific geographical locality. Future studies should apply this method and model to collect data from other countries and regions to verify the results and conduct comparative analyses to examine its applicability. Second, the choice of variables could be widened. We only tested the impact of internship satisfaction and the psychological contract on career identity behavior. Future research should add more variables based on this, and identify more factors that may influence career identity behavior, thereby enriching the theoretical literature in this area and providing improved guidance for practice.

## Data availability statement

The raw data supporting the conclusions of this article will be made available by the authors, without undue reservation.

## Ethics statement

Ethical review and approval were not required for the study on human participants in accordance with the local legislation and institutional requirements. Written informed consent from the patients/ participants or patients/participants legal guardian/next of kin was not required to participate in this study in accordance with the national legislation and the institutional requirements.

## Author contributions

YF: Conceptualization, Funding acquisition, Project administration, Supervision, Writing – review & editing. ZZ: Data curation, Formal analysis, Investigation, Methodology, Resources, Software, Validation, Visualization, Writing – original draft. XZ: Data curation, Funding acquisition, Investigation, Resources, Writing – review & editing. YL: Data curation, Formal analysis, Software, Validation, Writing – review & editing.

## References

[ref1] Argyris C. Ditz G. W. (1960). Understanding organizational behavior. Am. J. Sociol. 26, 133–458. doi: 10.2307/2090533

[ref2] Bal P. M. Chiaburu D. S. Jansen P. G. W. (2010). Psychological contract breach and work performance. J. Manag. Psychol. 25, 252–273. doi: 10.1108/02683941011023730

[ref3] Bal P. M. Kooij D. T. A. M. De Jong S. B. (2013). How do developmental and accommodative HRM enhance employee engagement and commitment? The role of psychological contract and SOC strategies. J. Manag. Stud. 50, 545–572. doi: 10.1111/joms.12028

[ref4] Baron R. M. Kenny D. A. (1986). The moderator mediator variable distinction in social psychological research: conceptual, strategic, and statistical considerations. J. Pers. Soc. Psychol. 51, 1173–1182. doi: 10.1037/0022-3514.51.6.1173, PMID: 3806354

[ref5] Bellini D. Cubico S. Ardolino P. Bonaiuto M. Mascia M. L. Barbieri B. (2022). Understanding and exploring the concept of fear, in the work context and its role in improving safety performance and reducing well-being in a steady job insecurity period. Sustain. For. 14:14146. doi: 10.3390/su142114146

[ref6] Bondt G. Vermeulen P. (2020). Business cycle duration dependence and foreign recessions. Scot. J. Polit. Econ. 68, 1–19. doi: 10.1111/sjpe.12261

[ref7] Bracho-Amador C. M. Granero-Gallegos A. Baena-Extremera A. López-García G. D. (2023). The effect of the motivational climate on satisfaction with physical education in secondary school education: mediation of teacher strategies in maintaining discipline. Behav. Sci. 13:178. doi: 10.3390/bs13020178 36829407 PMC9952606

[ref8] Cai Z. L. Zhu J. X. Tian S. Q. (2022). Preservice teachers' teaching internship affects professional identity: self-efficacy and learning engagement as mediators. Front. Psychol. 13:1070763. doi: 10.3389/fpsyg.2022.1070763, PMID: 36532965 PMC9748549

[ref9] Cerra V. Fatas A. Saxena S. C. (2023). Hysteresis and business cycles. J. Econ. Lit. 61, 181–225. doi: 10.5089/9781513536996.001

[ref10] Chacón-Cuberos R. Martínez-Martínez A. García-Garnica M. Pistón-Rodríguez M. D. Expósito-López J. (2019). The relationship between emotional regulation and school burnout: structural equation model according to dedication to tutoring. Int. J. Env. Res. Pub. He 16:4703. doi: 10.3390/ijerph16234703, PMID: 31779141 PMC6926892

[ref11] Chen T. L. Shen C. C. Gosling M. (2018). Does employability increase with internship satisfaction? Enhanced employability and internship satisfaction in a hospitality program. J. Hosp. Leis. Sport. To. 22, 88–99. doi: 10.1016/j.jhlste.2018.04.001

[ref12] Cheung R. Arnold J. (2014). The impact of career exploration on career development among Hong Kong Chinese university students. J. Coll. Stud. Dev. 55, 732–748. doi: 10.1353/csd.2014.0067

[ref13] Chisadza C. Clance M. (2021). Conflict heterogeneity in Africa. S. Afr. J. Econ. 89, 459–479. doi: 10.1111/saje.12297

[ref14] Coyle-Shapiro J. A. Coyle-Shapiro S. P. Coyle-Shapiro W. Chang C. (2019). Psychological contracts: past, present, and future. Annu. Rev. Organ. Psych. Organ. Behav. 6, 145–169. doi: 10.1146/annurev-orgpsych-012218-015212

[ref15] D'Abate C. P. (2009). Making the most of an internship: an empirical study of internship satisfaction. Acad. Manag. Learn. Educ. 8, 527–539. doi: 10.5465/AMLE.2009.47785471

[ref16] De Hauw S. De Vos A. (2010). Millennials’ career perspective and psychological contract expectations: does the recession lead to lowered expectations. J. Bus. Psychol. 25, 293–302. doi: 10.1007/s10869-010-9162-9

[ref17] De Vos A. Van der Heijden B. Akkermans J. (2020). Sustainable careers: towards a conceptual model. J. Vocat. Behav. 117:103196. doi: 10.1016/j.jvb.2018.06.011

[ref18] Dries N. Forrier A. De Vos A. Pepermans R. (2014). Self-perceived employability, organization-rated potential, and the psychological contract. J. Manag. Psychol. 29, 565–581. doi: 10.1108/JMP-04-2013-0109

[ref19] Dubey R. Altay N. Gunasekaran A. Blome C. Papadopoulos T. Childe S. J. (2018). Supply chain agility, adaptability and alignment: empirical evidence from the Indian auto components industry. Int. J. Oper. Prod. Manag. 38, 129–148. doi: 10.1108/IJOPM-04-2016-0173

[ref20] Fantinelli S. Galanti T. Guidetti G. Conserva F. Giffi V. Cortini M.. (2023). Psychological contracts and organizational commitment: the positive impact of relational contracts on call center operators. Adm. Sci. 13:112. doi: 10.3390/admsci13040112

[ref21] Fornell C. Larcker D. F. (1981). Structural equation models with unobservable variables and measurement error: algebra and statistics. J. Mar. Res. 18, 382–388. doi: 10.1177/002224378101800313

[ref22] Gligor D. M. Esmark C. L. Holcomb M. C. (2015). Performance outcomes of supply chain agility: when should you be agile? J. Oper. Manag. 33-34, 71–82. doi: 10.1016/j.jom.2014.10.008

[ref23] Granero-Gallegos A. López-García G. D. Baena-Extremera A. Baños R. (2023). Relationship between psychological needs and academic self-concept in physical education pre-service teachers: a mediation analysis. Sustain. For. 15:4052. doi: 10.3390/su15054052

[ref24] Gresse W. G. Linde B. J. (2020). The anticipatory psychological contract of management graduates: validating a psychological contract expectations questionnaire. S. Afr. J Econ. Manag. S. 23:a3285. doi: 10.4102/SAJEMS.V23I1.3285

[ref25] Hu L. Bentler P. M. (1999). Cutoff criteria for fit indexes in covariance structure analysis: conventional criteria versus new alternatives. Struct. Equ. Model. 6, 1–55. doi: 10.1080/10705519909540118

[ref26] Jung Y. M. (2020). Nursing students' career identity, satisfaction with major, and career stress by career decision type. Jpn. J. Nurs. Sci. 17:e12281. doi: 10.1111/jjns.12281, PMID: 31286671

[ref27] Kholaif M. M. N. H. Ming X. (2022). COVID-19’s fear-uncertainty effect on green supply chain management and sustainability performances: the moderate effect of corporate social responsibility. Environ. Sci. Pollut. R. 17, 1–22. doi: 10.1007/s11356-022-21304-9 PMC9201273 35708804

[ref28] Kline R. (2016). Principles and practice of structural equation modeling. NY, USA: The Guilford Press.

[ref29] Kock N. (2015). Common method bias in PLS-SEM: a full collinearity assessment approach. Int. J. E-Collab. 11, 1–10. doi: 10.4018/ijec.2015100101

[ref30] Li Y. (2002). Research on structure and related factors of employee psychological contract. [dissertation/master's thesis]. [Beijing]: Capital Normal University.

[ref31] Liu W. He C. Jiang Y. Ji R. Zhai X. (2020). Effect of gig workers' psychological contract fulfillment on their task performance in a sharing economy—a perspective from the mediation of organizational identification and the moderation of length of service. Int. J. Env. Res. Pub. He. 17:2208. doi: 10.3390/ijerph17072208, PMID: 32218336 PMC7177419

[ref32] Liu P. Ravenscroft N. Harder M. K. Dai X. (2016). The knowledge cultures of changing farming practices in a water town of the southern Yangtze Valley. China. Agr. Hum. Values. 33, 291–304. doi: 10.1007/s10460-015-9607-x

[ref33] Lodi E. Zammitti A. Magnano P. Patrizi P. Santisi G. (2020). Italian adaption of self-perceived employability scale: psychometric properties and relations with the career adaptability and well-being. Behav. Sci. 10:82. doi: 10.3390/bs10050082 32349212 PMC7287573

[ref34] Mahmood F. Ahmed Z. Hussain N. Zaied Y. B. (2022). Macroeconomic factors and financing strategies in working capital: evidence from China. Int. J. Financ. Econ. 6, 1–23. doi: 10.1002/ijfe.2666

[ref35] McDermott A. M. Conway E. Rousseau D. M. Flood P. C. (2013). Promoting effective psychological contracts through leadership: the missing link between HR strategy and performance. Hum. Resour. Manage-Us. 52, 289–310. doi: 10.1002/hrm.21529

[ref36] Mehta A. K. Thanki H. Panda R. Trivedi P. (2022). Exploring the psychological contract during new normal: construction and validation of the revised psychological contract scale. Int. J. Manpow. 9, 1–24. doi: 10.1108/IJM-05-2022-0201

[ref37] Mycos Research Institute. (2022). Employment report of Chinese college students in 2022. Beijing, China: Social Sciences Academic Press.

[ref38] Ng T. W. H. Feldman D. C. Lam S. S. K. (2010). Psychological contract breaches, organizational commitment, and innova-tion-related behaviors: a latent growth modeling approach. J. Appl. Psychol. 95, 744–751. doi: 10.1037/a0018804 20604593

[ref39] Obling A. R. (2022). Professional identity reconstruction: attempts to match people with new role expectations and environmental demands. Manag. Learn. 54, 468–488. doi: 10.1177/13505076211070906

[ref40] Podsakoff P. M. Mackenzie S. B. Lee J. Y. Podsakoff N. P. (2003). Common method biases in behavioral research: acritical review of the literature and recommended remedies. J. Appl. Psychol. 88, 879–903. doi: 10.1037/0021-9010.88.5.879 14516251

[ref41] Poppo L. Zhou K. Z. Li J. J. (2016). When can you trust ‘trust’? Calculative trust, relational trust, and supplier performance. Strateg. Manag. J. 37, 724–741. doi: 10.1002/smj.2374

[ref42] Praskova A. Creed P. A. Hood M. (2015). Career identity and the complex mediating relationships between career preparatory actions and career progress markers. J. Vocat. Behav. 87, 145–153. doi: 10.1016/j.jvb.2015.01.001

[ref43] Preacher K. J. Hayes A. F. (2004). SPSS and SAS procedures for estimating indirect effects in simple mediation models. Behav. Res. Methods Instrum. Comput. J. Psychon. Soc. Inc. 36, 717–731. doi: 10.3758/BF03206553, PMID: 15641418

[ref44] Qu H. Leung X. Y. Huang S. He J. (2021). Factors affecting hotel interns’ satisfaction with internship experience and career intention in China. J. Hosp. Leis. Sport. To. 28:100311. doi: 10.1016/j.jhlste.2021.100311

[ref45] Rayton B. A. Yalabik Z. Y. (2014). Work engagement, psychological contract breach and job satisfaction. Int. J. Hum. Resour. Manag. 25, 2382–2400. doi: 10.1080/09585192.2013.876440

[ref46] Robinson S. L. Rousseau K. D. M. (1994). Changing obligations and the psychological contract: a longitudinal study. Acad. Manag. J. 37, 137–152. doi: 10.2307/256773

[ref47] Robinson S. L. Wolfe Morrison E. (2000). The development of psychological contract breach and violation: a longitudinal study. J. Organ. Behav. 21, 525–546. doi: 10.1002/1099-1379(200008)21:53.0.CO;2-T

[ref48] Rogozińska-Pawełczyk A. Gadomska-Lila K. (2022). The mediating role of organisational identification between psychological contract and work results: an individual level investigation. Environ. Res. Public Health 19:5404. doi: 10.3390/ijerph19095404, PMID: 35564799 PMC9099778

[ref49] Rousseau D. M. (1989). Psychological and implied contracts in organizations. Employ. Responsib. Rig. 2, 121–139. doi: 10.1007/BF01384942

[ref50] Rousseau D. M. (1995) Psychological contracts in organizations: Understanding written and unwritten agreements. Thousand Oaks, CA, USA: Sage.

[ref51] Rousseau D. M. (2004). Psychological contracts in the workplace: understanding the ties that motivate. Acad. Manag. Perspect. 18, 120–127. doi: 10.5465/ame.2004.12689213

[ref52] Sánchez-Sellero M. C. Sánchez-Sellero P. Cruz-González M. M. Sánchez-Sellero F. J. (2017). Stability and satisfaction at work during the Spanish economic crisis. Prague. Econ. Pap. 26, 72–89. doi: 10.18267/j.pep.596

[ref53] Seyitoğlu F. Yirik S. (2014). Internship satisfaction of students of hospitality and impact of internship on the professional development and industrial perception. Asia. Pac. J. tour. Res. 20, 1414–1429. doi: 10.1080/10941665.2014.983532

[ref54] Shen Y. M. Schaubroeck J. M. Zhao L. Wu L. (2019). Work group climate and behavioral responses to psychological contract breach. Front. Psychol. 10:67. doi: 10.3389/fpsyg.2019.00067, PMID: 30778308 PMC6369362

[ref55] Shih C.-y. (2014). Relations and balances: self-restraint and democratic governability under Confucianism. Pac. Focus. 29, 351–373. doi: 10.1111/pafo.12034

[ref56] Shrout P. E. Bolger N. (2002). Mediation in experimental and nonexperimental studies: new procedures and recommendations. Psychol. Methods 7, 422–445. doi: 10.1037/1082-989X.7.4.422, PMID: 12530702

[ref57] Simbula S. Margheritti S. Avanzi L. (2023). Building work engagement in organizations: a longitudinal study combining social exchange and social identity theories. Behav. Sci. 13:83. doi: 10.3389/fpsyg.2021.679490, PMID: 36829312 PMC9952149

[ref58] Simons J. D. (2021). From identity to enaction: identity behavior theory. Front. Psychol. 12:679490. doi: 10.31234/osf.io/5r2vx, PMID: 34504457 PMC8423104

[ref59] Stuer D. De Vos A. Van der Heijden B. I. J. M. Akkermans J. (2019). A sustainable career perspective of work ability: the importance of resources across the lifespan. Int. J. Env. Res. Pub. He. 16:2572. doi: 10.3390/ijerph16142572, PMID: 31323860 PMC6678940

[ref60] Suazo M. M. Turnley W. H. (2020). Perceived organizational support as a mediator of the relations between individual differences and psychological contract breach. J. Manag. Psychol. 25, 620–648. doi: 10.1108/02683941011056969

[ref61] Taylan O. Alkabaa A. S. Ylmaz M. T. (2022). Impact of Covid-19 on G20 countries: analysis of economic recession using data mining approaches. Financ. Innov 8:81. doi: 10.1186/s40854-022-00385-y, PMID: 36091580 PMC9441845

[ref62] Tomprou M. Rousseau D. M. Hansen S. D. (2015). The psychological contracts of violation victims: a post-violation model. J. Organ. Behav. 36, 561–581. doi: 10.1002/job.1997

[ref63] Topa G. Aranda-Carmena M. De-Maria B. (2022). Psychological contract breach and outcomes: a systematic review of reviews. Int. J. Env. Res. Pub. He. 19:15527. doi: 10.3390/ijerph192315527, PMID: 36497602 PMC9737235

[ref64] Tortorella G. L. Giglio R. Dun D. H. (2019). Industry 4.0 adoption as a moderator of the impact of lean production practices on operational performance improvement. Int. J. Oper. Prod. Manag. 39, 860–886. doi: 10.1108/IJOPM-01-2019-0005

[ref65] Trede F. Macklin R. Bridges D. (2012). Professional identity development: a review of the higher education literature. Stud. High. Educ. 37, 365–384. doi: 10.1080/03075079.2010.521237

[ref66] Tsui A. S. Pearce J. L. Porter L. W. Tripoli A. M. (1997). Alternative approaches to the employee-organization relationship: does investment in employees pay off. Acad. Manag. J. 40, 1089–1121. doi: 10.2307/256928

[ref67] Turnley W. (2003). The impact of psychological contract fulfillment on the performance of in-role and organizational citizenship behaviors. Aust. J. Manag. 29, 187–206. doi: 10.1016/S0149-2063(02)00214-3

[ref68] Wang X. Binti Omar N. A. (2023). Nexus between brand love, loyalty, affective commitment and positive word of mouth: in the context of social identity theory. Sustain. For. 15:3813. doi: 10.3390/su15043813

[ref69] Warwick M. K. David V. (2020). Global macroeconomic cooperation in response to the covid-19 pandemic: a roadmap for the G20 and the IMF. Oxford. Rev. Econ. Pol. 36, S297–S337. doi: 10.1093/oxrep/graa032 PMC7499757 40504204

[ref70] Weiss J. K. Bottling M. Kaerner T. (2023). Professional identification in the beginning of a teacher's career: a longitudinal study on identity formation and the basic psychological need for autonomy in VET teacher training. Front. Psychol. 14:1196473. doi: 10.3389/fpsyg.2023.1196473, PMID: 37599718 PMC10434542

[ref71] Willetts G. Clarke D. (2013). Constructing nurses’ professional identity through social identity theory. Int. J. Nurs. Pract. 20, 164–169. doi: 10.1111/ijn.12108 24713013

[ref72] Xu Y. Cheng Z. Zhang Y. (2021). Promotion of millennial employees' well-being in China based on organizational career management. Soc. Behav. Personal. 49:e9572, 1–12. doi: 10.2224/sbp.9572

[ref73] Yang J. Liu Y. Kholaif M. M. N. H. K. (2022). The impact of relationship management on manufacturer resilience in emergencies. Kybernetes 12, 1–30. doi: 10.1108/K-08-2022-1198

[ref74] Yang J. Liu Y. Kholaif M. M. N. H. K. (2023). Trust relationship with suppliers, collaborative action, and manufacturer resilience in the covid-19 crisis. Behav. Sci. 13:33. doi: 10.3390/bs13010033, PMID: 36661605 PMC9854493

